# What Is Thought of Our National Organization on the Other Side of the Atlantic

**Published:** 1855-12

**Authors:** 


*What is thought of our National Organization on the other side of the Atlantic.*— The *Medical News* of Philadelphia, copies the following complimentary notice of the American Medical Association and its Transactions, from the Association Medical Journal of Great Britain. Let the profession in America continue to sustain their National Organization in such a manner that it shall constitute an example well worthy of imitation by our brethren on the other side of the wide waters.

"Of such a body as this—the most enlightened representatives of the greatest medical constitutency in the world, it would be presumptuous for us to speak in terms of praise; and we must be content to place before our readers a sketch of the scope and result of its past labors.

"We find then, on referring to the volumes of *Transactions* already published by the Association, that, in the first place, the duties of the standing committees have been ably and thoroughly fulfilled. We find the progress of medical science as a whole, and of practical medicine, surgery, and obstetricy, the most prominent divisions of the main subject, have been carefully and accurately traced in a series of reports worthy of the highest praise. Clear, concise, and comprehensive, these reports reflect the greatest credit, not only upon the committees from whom they have eminated but also upon the judgement of the Association, in selecting the members of those committee for their respective duties.

"The Committee on Medical Education, having commenced its labors by a description of melancholy deficiencies, and having constantly endeavored to remove them, was enabled to sum up its last report in the following words of cheerfulenss and encouragement:—

" Your Committee are pleased to bring, from the different sections of the country inwhich they reside, gratifying assurances that the labors of this Association have thus far been fruitful of good results. The volutary principle in its organization, and its representative character, increases from year to year its moral power and influence, which have been manifest in the increase of State and county societies, and in the efforts which many of the medical schools have made to conform to its recommendations.'

" The Committee on Medical Literature has ' considered the general subject assigned to it, under three heads, viz.: the medica periodicals; the medical publications, including monographs and books; and the best means of elevating the character and extending the usefulness of the national medical literature.' The first report intimates (it is a remarkable fact that, in the New World the errors of medical periodicals are not ' beneath the dignity' of a learned society) that, with regard to the medical journals, ' a sore cause of complaint, of occasional but not frequent occurrence, is to be found in the liberties allowed to anonymous writesr—not so much with regard to each other, for, if "Medicus" and " Senex" were to succeed in reciprocal annihilation, the loss might not be serious—but with regard to their neighbors at large, and to things in general.' ' An editor,' says the report, ' is responsible that nothing shall be admitted into his pages, the essential character of which is hostile and inflammatory, on the same principle that he is bound to be courteous in his common intercourse.' In the same paper the American translations or reprints, together with the original works on medicine, are criticized or mentioned. Under the surveillance of a committee guided by the principles we have described, it will afford no matter of surprise that the report on Medical Literature presented in 1853, should contain much to indicate progress as well as much of warning and admonition for the future. Although very sparing of praise, it concludes by the expression of a ' confident conviction that the literature of our profession has already commenced a rapid improvement, which is destined to continue until it attains an elevation and influence corresponding with the high social and political destiny of our country.'

' Besides the regular proceedings of the standing committee, the American Association has accomplished a laborious investigation of the indigenous Medical Flora of the Union; has examined into, and reported upon, the adulteration of drugs; has ascertained the sanitary condition of the various States, and the difference between them in respect of the public health; and has on several occasions appointed committees for the study of epidemics and of special scientific subjects. These committees have collected and published a vast amount of highly valuable information.

"We have now, as fully as our narrow limits will permit, laid before our readers an account of the government, the animus, and the operations of the American Medical Association. It would be a pleasant task to dwell longer upon these topics, and to fill in with appropriate shading the scant outline that now appears upon our pages. But instead of doing so, we must turn at once to the practical question which even this outline cannot fail to suggest; we must inquire into the causes of that success, especially in ethical reform, which we cannot fail greatly to admire, and can scarcely refrain from envying. Those causes are to be found, we believe, solely in the moral power which is inseparable from a constitution based upon the principles of equal representation."

